# Obituary

**Published:** 1894-03

**Authors:** 


### Obituary.

Dr. Theodor Billroth, of Vienna, died February 5, 1894, of heart disease. He was one of the most celebrated surgeons of his day and was an author of great renown. He continued his surgical and literary work almost to his end. He published a work on Pathological Histology in 1858; and another on Classification, Diagnosis and Prognosis of Tumors, translated in 1861, through which he became known to the profession in this country. His lectures on Surgical Pathology, published in 1872 by the Appletons, became a text-book in most medical colleges in America. It is justly claimed that his most striking surgical achievement was the performance of the first successful resection of the pylorus for malignant disease, Pean had previously done the operation without success.

